# Postoperative pain after cesarean section: assessment and management in a tertiary hospital in a low-income country

**DOI:** 10.1186/s12913-019-3911-x

**Published:** 2019-01-25

**Authors:** Andrew Kintu, Sadiq Abdulla, Aggrey Lubikire, Mary T. Nabukenya, Elizabeth Igaga, Fred Bulamba, Daniel Semakula, Adeyemi J. Olufolabi

**Affiliations:** 1 Department of Anesthesia, Makerere University College of Health Sciences, Mulago, Uganda; 2 Department of Anesthesia, University of British Columbia and BC Women’s Hospital, Vancouver, Canada; 3 Department of Anesthesia, Busitema University Faculty of Health Sciences, Mbale, Uganda; 4 Innovations and Knowledge Translation Office, Makerere University College of Health Sciences, Mulago, Uganda; 5 Department of Anesthesia, Duke University, Durham, NC USA

**Keywords:** Cesarean section, VAS, Postoperative pain, Post-caesarean section pain, Mulago hospital

## Abstract

**Background:**

There is little information about the current management of pain after obstetric surgery at Mulago hospital in Uganda, one of the largest hospitals in Africa with approximately 32,000 deliveries per year. The primary goal of this study was to assess the severity of post cesarean section pain. Secondary objectives were to identify analgesic medications used to control post cesarean section pain and resultant patient satisfaction.

**Methods:**

We prospectively followed 333 women who underwent cesarean section under spinal anesthesia. Subjective assessment of the participants’ pain was done using the Visual Analogue Scale (0 to 100) at 0, 6 and 24 h after surgery. Satisfaction with pain control was ascertained at 24 h after surgery using a 2-point scale (yes/no). Participants’ charts were reviewed for records of analgesics administered.

**Results:**

Pain control medications used in the first 24 h following cesarean section at this hospital included diclofenac only, pethidine only, tramadol only and multiple pain medications. There were mothers who did not receive any analgesic medication. The highest pain scores were reported at 6 h (median: 37; (IQR:37.5). 68% of participants reported they were satisfied with their pain control.

**Conclusion:**

Adequate management of post-cesarean section pain remains a challenge at Mulago hospital. Greater inter-professional collaboration, self-administered analgesia, scheduled prescription orders and increasing availability of analgesic drugs may contribute to improved treatment of postoperative pain with better pain scores.

## Background

Inadequately treated postoperative pain can contribute significantly to morbidity of surgical patients, resulting in the delay of patients’ recovery and ability to return to daily functional activities [1]. Early recovery is especially important for a patient who is expected to take care of her newborn shortly after an operative procedure. Evidence from studies done in high income settings has demonstrated that inadequately treated pain after cesarean section is associated with an increased incidence of chronic pain [2] and post-traumatic stress syndrome [3].

In low-income countries, postoperative pain management can be particularly challenging for several reasons including the expectation of postoperative pain by patients (thereby making no effort to request for pain relief) and the high patient-to-nurse ratio that limits assessment of pain and administration of adequate pain relief medication [4]. At Mulago National Referral Hospital, a tertiary teaching hospital, it has been shown that patients have inadequate pain control after orthopedic and general surgery [5], but little is known about the postoperative pain management after obstetric surgery. We hypothesized that women undergoing cesarean section do not have satisfactory postoperative pain management in the first 24 h after surgery.

The primary goal of the study was to determine the current pain status and management following cesarean section at Mulago National Referral Hospital, Kampala, Uganda. The secondary objectives were to determine patient satisfaction with their pain control and document the methods and regimes of analgesia used.

## Methods

We conducted a prospective descriptive hospital based survey. The study was conducted in the obstetric ward at Mulago National Referral Hospital in Kampala, Uganda between November 2014 and January 2015. Mulago National Referral hospital was built with a 1500 bed capacity but currently functions as 2500 bed capacity with approximately 32,000 deliveries a year, which includes a 20% cesarean delivery rate.

### Study population

Eligible participants included all parturients undergoing cesarean section between 0800 and 1800 h, under spinal anesthesia with an American Society of Anesthesiologists physical status classification (ASA) of I to III and able to communicate freely with a non-family member interpreter to obtain informed consent. All parturients who experienced failed spinal anesthesia and required general anesthesia were excluded from the study.

### Sampling

For purposes of continued and easy patient follow up, we only screened parturients whose cesarean section were performed between 0800 and 1800 h due to availability of staff responsible for consenting patients. Consecutive sampling was used to recruit the parturients into the study.

We calculated an initial sample size of 245 participants using the Kish and Leslie formula (1965) for a single proportion based on a previous study by Ismail et al. [6] for a power of 80% at 95% confidence. We adjusted this sample size by an additional 35% to a total of 333 to compensate for any potential loss to follow-up and non-response, which we assumed would be high because of the level of activity around postoperative patients with newborns.

### Ethical approval

Approval for this study was granted by the Mulago Hospital Research and Ethics Committee. Written informed consent was obtained from parturients with or without the help of an interpreter and was certified by either the patient’s signature or thumbprint.

### Study procedure

We screened 457 patients in the anesthesia waiting area in the obstetric unit at Mulago Hospital and recruited 333 patients who were scheduled for both elective and emergency cesarean section under neuraxial anesthesia using hyperbaric bupivacaine 10 to 12 mg. 124 patients did not meet inclusion criteria due to refusal to consent, failed regional technique or the occurrence of CS late in the day. Patient data were obtained from interviewing patients, reviewing medical charts, and nurses’ patient records. The information was recorded in a pre-tested questionnaire and transferred into an electronic database. Personal identification information was replaced with anonymous participant number to mark the questionnaires. Participants who met study criteria had their pain assessed using the visual analogue scale (VAS) (using a 0 to 100 scale) with 0 having no pain and 100 having experienced the worst pain. The pain VAS was self-completed by the patient who was asked to place a line perpendicular to the VAS line at the point that represented their pain intensity. Using a foot ruler, the pain score was determined by measuring the distance on the 100 line between the no pain anchor and the patient’s mark, providing a range of scores from 0 to 100. Pain category was determined as 0–4 no pain, 5–44 mild pain, 45–74 moderate pain, and 75–100 severe pain. Pain was assessed as soon as the patient arrived at the recovery area (0 h) and at 6 h and at 24 h in the postoperative obstetric ward.

In addition, at 24 h, all participants were asked to provide a yes or no response regarding their satisfaction with pain control. A record of all analgesics administered in the first 24 h after surgery were documented including the time of administration, name of analgesic and prescriber. During the entire study period, the study team did not interfere with pain management of the participants.

### Variables and measurements

Independent variable was the analgesic treatment group while the dependent variable was VAS scores at the different times of assessment.

### Statistical analysis

The data were exported to Stata statistical analysis software version 12.1 for analysis. Our analysis is based on data from all the 333 participants enrolled in the study. Although we noted missing data on different variables, we determined it would be best not t to exclude any participant with missing data but include all in the final analysis. Because of this, some variables have different totals.

For patient characteristics, we present frequencies and proportions. For the primary outcome, we summarized findings at the various time points using medians and interquartile range (IQR).

For secondary objectives, we calculated the proportion of participants in the different treatment groups. In addition, we assessed differences in pain scores among the different treatment groups and conducted a post-hoc evaluation for pairs of treatment groups found to have statistically significant differences in pain scores. Assessment of normality was done using graphical plots and the Shapiro-Wilk test. Given that the data were not normally distributed we used the Kruskal-Wallis test to determine differences in the pain scores among the treatment groups. The data from this analysis is presented as median pain scores, and their IQR. For patient satisfaction, a proportion of participants satisfied with their analgesia was estimated.

## Results

We recruited 333 parturients in the anesthesia waiting area in the obstetric unit. 43 participants had at least one missing variable data. All participants were included in the analysis. For clarity, we state the number of participants with complete data for each outcome.

The baseline characteristics of the study participants and care providers are summarized in Table 1. Majority of participants were 30 years or younger with at least two previous pregnancies carried beyond 28 weeks of gestation. The commonest indications for cesarean delivery were previous cesarean section scar and obstructed labor. In this cohort, 72% (237/328) of anesthesia was provided by the anesthetic officer (non-physician provider). For analgesic prescription, complete data was available for 290 participants. Analgesia was prescribed by surgeons for 95% of the parturients (275/290). No analgesic medication was prescribed by a nurse. Only 42% (123/290) of participants received their analgesics as prescribed (Table 1). In all participants, the median time to first analgesic administration after leaving the operating room was 241 min (IQR: 350). At 24 h assessment, 68% (197/290) of participants determined pain control was satisfactory.

**Table 1:** Baseline characteristic, indications for cesarean section, anesthesia providers, prescribing staff and patients whose drugs were administered as prescribed

Characteristics (N)		Frequency (n)	Percentage
Age (*N* = 326)	≤20	65	20.0
	21–30	198	61
	≥31	63	19
Parity* (*N* = 325)	First time pregnancy	96	30
	2	102	31
	≥3	127	39
Common indications for cesarean section**			
Fetal distress^#^ (*N* = 333)	No	301	90
	Yes	32	10
Obstructed labor*** (*N* = 333)	No	244	23
	Yes	89	27
Previous C-section (*N* = 333)	No	210	63
	Yes	123	37
Big baby**** (*N* = 333)	No	309	93
	Yes	24	7
Prolonged labor^##^ (*N* = 333)	No	311	93
	Yes	22	7
Care practices			
Anesthesia provider (*N* = 328)	^@^Senior house officer	57	18
	^@@^Anesthetic officer	237o	72
	Anesthesiologist	34	10
Prescribing staff (*N* = 328)	Surgeon	310	95
	Anesthetic provider	18	5
Patients whose Drugs were administered as prescribed (*N* = 297)	No	**173**	**58**
	Yes	**124**	**42**

Key: *Number of pregnancies carried above 28 weeks, **Most of the patients had more than one indication for cesarean section, ***Obstructed labor referred to failure of labor to progress (no change in cervical dilation and descent of fetus) despite good uterine contractions for more than 4 h, ****Big baby was any fetus ≥4kgs diagnosed by ultra sound or clinical examination, ^#^Fetal distress referred to any fetal heart rate below 120 or above 160, ^##^Prolonged labor referred to any patient in active labor (cervical dilation greater than 4) for ≥14 h, ^@^Senior House officer (SHO) is a Masters of Medicine in anesthesia trainee (also referred to as a resident), ^@@^Anesthetic Officer a non-physician anesthesia provider with a diploma training in anesthesia, ^@@@^Anesthesiologist is a physician who has completed Masters of Medicine in anesthesia training

**Fig. 1:**
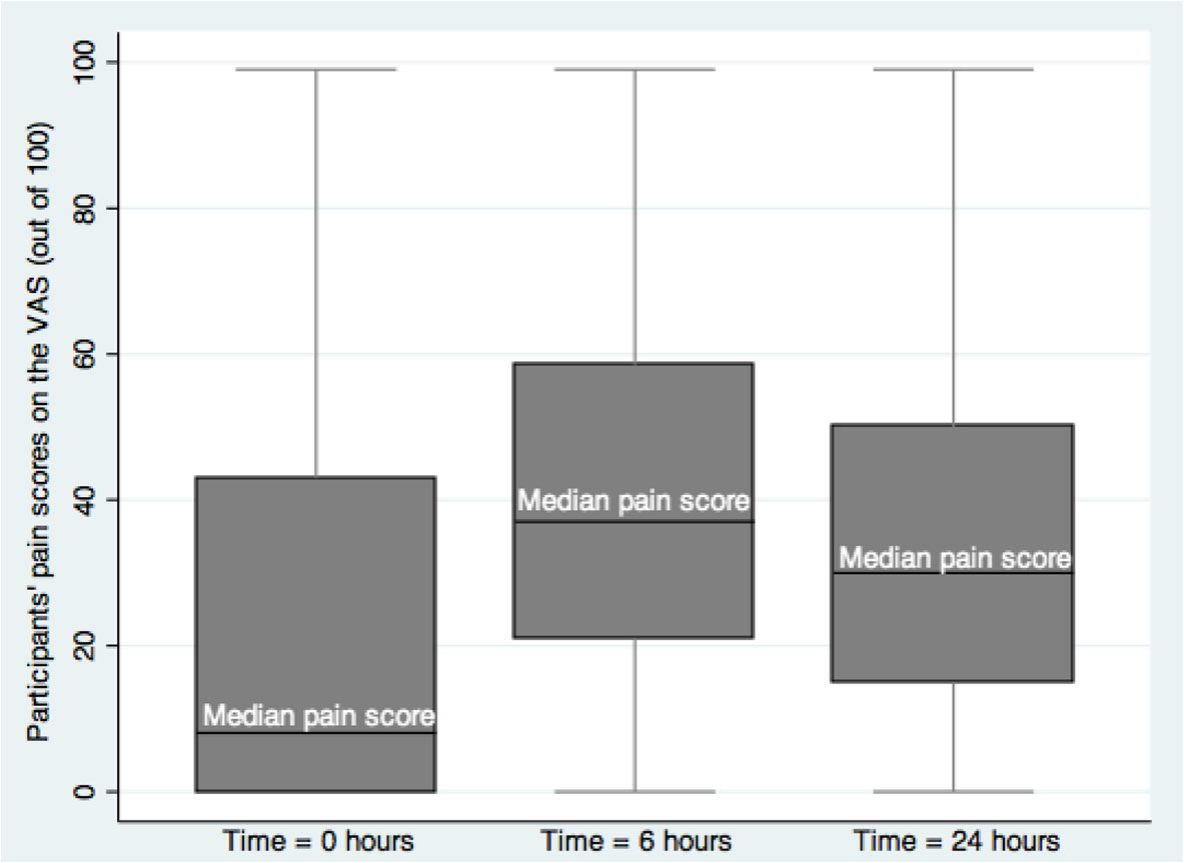
A comparative distribution of pain scores in the first 24 h after C-section

Figure 1 shows the comparison of the distribution of pain scores at the different time points. For this outcome, data was complete for all participants (333) at T0 hours and T6 hours but for only 301 participants at T24 h. The median pain scores increased from 8 (IQR: 43) at T0 hours to 37 (IQR: 37.5) at T6 hours and reduced to 30 (IQR: 35) at T24 h.

At all the three time points, a higher proportion of participants experienced no pain or mild pain. However, this proportion reduced markedly between T0 hours 77% (257/333) and T6 hours 61% (203/333) and increased at T24 h 71% (214/301).

On the other hand, the proportion of participants experiencing severe pain increased from 11% (37/333) at T0 to 14% (48/333) at T6 and then reduced to 6% (18/301) at T24 hours. The trend was similar for participants who experienced moderate pain.

In the first 24 hours following surgery, 44% (144/327) participants received only one type of analgesic drug, 14% (47/327) received multiple drugs while 42% (136/327) received none. Intramuscular diclofenac was the most prescribed analgesic followed by tramadol and pethidine respectively. No patient received intravenous or intramuscular morphine.

4% (13/328) participants who had complete data on anesthesia technique received intrathecal opioids (morphine) as part of the spinal anesthetic for C- section.

Participants were classified into six treatment groups according to the analgesic medication they received during the study period. These groups included: diclofenac only, pethidine only, intrathecal opioid only, tramadol only, multiple pain medications, and no pain medication. The corresponding proportions at T0 h, T6 h and T 24 h are shown in Table 2.

**Table 2:** Management of post cesarean section pain in the first 24 h

Treatment group	T0 (*N* = 319)		T6 (*N* = 311)		T24 (*N* = 296)	
	n	%	n	%	n	%
Diclofenac only	80	25	77	25	76	26
Pethidine only	23	7	23	7	21	7
Intrathecal morphine only	3	1	3	1	3	1
Tramadol only	35	11	34	11	31	10
Multiple analgesics^a^	46	15	45	15	43	15
No analgesics	132	41	129	41	122	41

^a^Multiple analgesics included anyone who received more than one analgesic drugs, of any kind

**Fig. 2:**
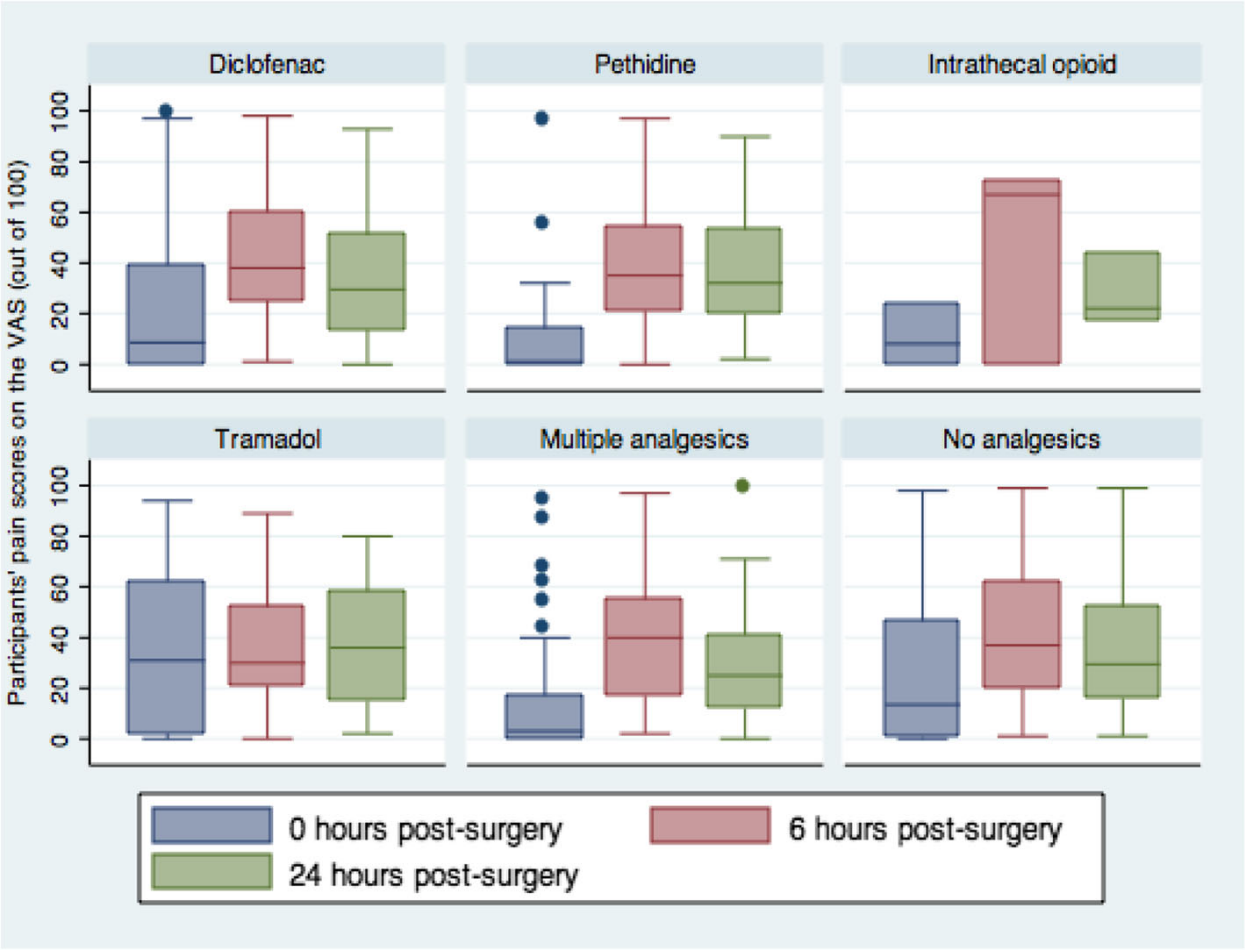
A box and whisker plot showing the distribution of median VAS scores among groups of treatment at T0, T6, T24

At T0 hours, the median pain scores were lowest for the group that received pethidine only (1, IQR:14) and the group that received a combination of analgesics (3, IQR:17) while the group that received tramadol only had the highest pain scores (31, IQR:60). At T6, the median pain scores were lowest for the group that received tramadol only (30, IQR:31) while the group that received intrathecal morphine had the highest pain scores (67, IQR:72). At T24, the median pain scores were lowest for the group that received intrathecal morphine (22, IQR:27) while the group that received only tramadol had the highest pain scores (36, IQR:43) (Fig. 2).

The distribution of the pain scores in the different treatment groups at all the three time points did not follow a Gaussian distribution. The Kruskal-Wallis equality-of-populations rank test for the pain scores at time = T0 revealed that at least one treatment group had median pain scores that were statistically significantly different from those of participants in other groups (X^2^ (5df) =16.15; *p* = 0.006). A further post-hoc analysis using Dunn’s pairwise comparison of treatment groups at time T0, adjusting for multiple comparisons using the Bonferroni method revealed that participants on pethidine had statistically significantly lower pain scores than those on tramadol (Z = − 3.13, *p* = 0.01) or those who did not receive analgesics (Z = − 2.78, *p* = 0.04), while those who received multiple types of analgesics had lower pain scores than those who received tramadol (Z = 2.9, *p* = 0.028). However, there was no statistically significant difference in the median pain scores between the rest of the treatment group pairs for time T0; and no statistically significant difference in the median pain scores among the different treatment groups at time T6 (X^2^(5df) =1.8, *p* = 0.87) and T24 (X^2^(5df) =2.2, *p* = 0.81).

## Discussion

This study is an exploratory survey examining the experience of post C-section pain in patients at a large tertiary hospital in Uganda. It highlights the gaps in pain management but also the opportunity for improvement. Many of the participants in this study did not receive their analgesics as prescribed (42%). A considerable number did not get any analgesia, similar to the practice in general surgery patients in the same hospital as well as in some hospitals in Nigeria [5, 7]. In the end, only 68% reported being satisfied with their pain control. This discrepancy between prescription and actual administration of analgesics may be due to irregular and inadequate availability of medications due to hospital stock out leading to patients having to purchase their own drug, high patient: nurse ratio, poor prescription practices and unfamiliarity with some modern analgesic techniques and drugs [4]. The high patient: nurse ratio makes postoperative pain management challenging in many low-income countries. These findings are consistent with the results of Kiswezi et al, that showed inadequate post-laparotomy pain management in the same hospital [5]. In that study, factors that contributed to poor pain management included inconsistent pain assessment and irregular supplies of prescribed drugs. Prescribing orders could also play a role as many doctors write *pro re nata* (prn) orders rather than scheduled orders. Inadequate postoperative pain control seem a universal surgical problem demonstrated in other low-income and high-income countries [8–10].

The types of analgesics that were used are similar to those used in other low-income settings for obstetric patients [7, 11]. In the first 24 h after C-section, the largest number of participants received only intramuscular diclofenac, followed by multiple drugs and only tramadol in that order. The most popular route of administration in our cohort was the intramuscular route. Studies have shown that use of single analgesics is not effective in the management of moderate and severe pain [12, 13]. Multimodal analgesia is currently the recommended practice [14, 15] although median pain scores for participants who received single analgesic were comparable to those in the multiple analgesic category across the three time points, in our study. Our study also suggests that different types of analgesic drugs may be better at different time points. For example, Pethidine seem adequate analgesic if administered immediately following surgery while tramadol may be better if administered 4–6 h after surgery. Patients who received intrathecal morphine had better pain control towards the end of 24 hours. This however will need to be explored locally in future studies.

Many high-income countries use longer acting intrathecal opioids that reduce postoperative pain in the first 24 hours. Despite the benefit of intrathecal morphine towards longer analgesic effects for cesarean sections [16, 17], its use during the study period was minimal possibly due to unfamiliarity of use by anesthesia providers. Irregular availability of opioids, persistent concern of the risk of respiratory depression and the inability to monitor patients limit their use in low-income settings. The worst pain experienced in our study population was 6 hours post- C-section likely due to spinal anesthesia wearing off without further analgesia given as shown by the time it took for participants to receive analgesic drugs after leaving the operating room (241 min (IQR 350, range 0–950). This breakthrough pain experience is not usually experienced with intrathecal morphine use. Intrathecal morphine was introduced into practice at Mulago hospital during the study period and supplies were irregular. As of 2018, the use of intrathecal morphine at this hospital has markedly increased.

Finally, surgeons accounted for 95% of the postoperative analgesia prescriptions. This practice is similar in other low-middle income countries [4, 6, 9, 11]. In high-income countries, immediate postoperative analgesia is managed by anesthesia or acute pain services that are experienced and trained in the use of prescribed analgesia. We would suggest that this practice needs to be reviewed in the light of persistent failure to adequately manage postoperative surgical pain.

A limitation in our study is not exploring other factors that influence pain such as cultural and personal perceptions of staff and patients.

## Conclusion

The management of post cesarean section pain in Mulago hospital is inadequate demonstrated by significant number of participants who received no analgesic medication after leaving the operating room and this needs addressing. The majority of participants also did not receive treatment as prescribed and overall level of satisfaction with pain management is relatively low. Awareness and practice change need to occur. Use of multimodal analgesia would be beneficial in our setting, and as such should be encouraged. Interprofessional collaboration, training in pain and pain control, ensuring availability of analgesic drugs, and self-administered analgesia may contribute to improvements in patients’ pain experience following post C-section at Mulago hospital.

## Abbreviations

C- section: Cesarean section
df: Degrees of freedom
h: Hours
IQR: Interquartile range
mg: Milligrams
min: Minutes
mm: Millimeters
T: Time
VAS: Visual analogue scale
